# Opioid-induced bowel dysfunction: suggestions from a multidisciplinary expert Board

**DOI:** 10.1007/s00520-019-04688-2

**Published:** 2019-02-18

**Authors:** Marco Rossi, Giuseppe Casale, Danilo Badiali, Federica Aielli, Maria Antonietta Aloe Spiriti, Roberto Arcioni, Francesca Bordin, Maurizio Ferrara, Gloria Morelli Sbarra, Antonio Corcione, Franco Marinangeli, Paolo Marchetti

**Affiliations:** 1 Department of Anaesthesia and Emergency Medicine—IRCCS Fondazione Policlinico Universitario A. Gemelli, Università Cattolica del Sacro Cuore, Largo Agostino Gemelli, 8, 00168 Rome, Italy; 2 Antea Associazione ONLUS, Rome, Italy; 3 Internal Medicine and Medical Specialties, Sapienza University of Rome, Rome, Italy; 4 Department of Applied Clinical Sciences and Biotechnology, University of L’Aquila, L’Aquila, Italy; 5 Department of Clinical and Molecular Medicine, Azienda Universitaria Ospedaliera Sant’Andrea, Rome, Italy; 6 Department of Anaesthesia and Intensive Care, Division of Pain Therapy, Azienda Ospedaliera Universitaria Sant’Andrea, Rome, Italy; 7 Palliative Care Unit, Hospice INI, Istituto Neurotraumatologico Italiano, Grottaferrata, Rome, Italy; 8 Multidisciplinary Department of Anaesthesia and Post-Operative Intensive Care, AORN—Ospedali dei Colli-Monaldi, Naples, Italy; 9 Department of Life, Health and Environmental Sciences, University of L’Aquila, L’Aquila, Italy; 10 Medical Oncology, Sapienza University of Rome and IDI-IRCCS of Rome, Rome, Italy

**Keywords:** Opioids, Constipation, Mu receptor, Pain, Multidisciplinary

## Abstract

Constipation, one of the adverse effects of opioid therapy with a major impact on quality of life, is still an unmet need for cancer patients, particularly those with an advanced and progressive disease, and for non-cancer patients chronically treated with opioids. The awareness of this condition is poor among healthcare providers, despite the recent publication of guidelines and consensus conferences. An early multidisciplinary approach of opioid-induced bowel dysfunction (OIBD), based on available therapies of proven effectiveness, could support clinicians in managing this condition, thus increasing patients’ adherence to pain therapy. Several Italian experts involved in the management of patients suffering from pain (anaesthesia pain therapy, oncology, haematology, palliative care, gastroenterology) joined in a Board in order to draw up an expert opinion on OIBD. The most frequent and still unsolved issues in this field were examined, including a more comprehensive definition of OIBD, the benefits of early intervention to prevent its occurrence and the most appropriate use of peripherally acting mu-opioid receptor antagonists (PAMORAs). The use of the recently introduced PAMORA naloxegol was analysed, in light of the current literature. The Board proposed a solution for each open issue in the form of recommendations, integrated with the contribution of representatives from different disciplines and often accompanied by procedural algorithms immediately usable and applicable in daily clinical practice. Safety and quality of life of the patient suffering from pain and from the adverse effects of pain therapies have been the mainstays of this expert opinion, in cooperation with general practitioners and caregivers.

## Introduction

The optimal management of opioid therapy–induced adverse effects is still an unmet need. Constipation is a considerable issue for cancer patients, particularly those with a progressive disease [1]. An accurate analysis of the appropriateness of current therapies requires involvement of the various healthcare professionals who provide care to patients on opioid therapy.

An early multidisciplinary approach may support clinicians in the management of opioid-induced constipation (OIC), minimise the risks of neglecting it and increase the adherence of patients to the proposed pain therapy.

A panel of Italian clinicians from different areas of expertise (anaesthesiology and pain therapy, oncology, haematology, palliative care, gastroenterology) convened to draw up an expert opinion on OIC, aiming at constituting a first step towards a shared pathway among the various clinical disciplines and hopefully to involve nursing professionals and caregivers.

### The palliative care physician’s point of view

Palliative care networks show different organisational models, generally developed on the experience of each group. Such fragmentation may affect care, raising the risk of inadequate treatment. The management of constipation requires an interdisciplinary approach, especially in palliative care patients. For patients with advanced disease, the most common causes of constipation are reduced physical activity, being bedridden, dehydration, loss of autonomy in bowel evacuation, malnutrition and indeed the use of opioids.

## A terminology issue?

The definition of OIC is currently based on four parameters [2]:

- reduced bowel movement frequency;
- development or worsening of straining to pass bowel movements;
- a sense of incomplete rectal evacuation;
- harder stool consistency.

The term opioid-induced bowel dysfunction (OIBD) is preferred over OIC as it implies a broader definition involving the entire gastrointestinal apparatus and refers to a wider range of parameters, including pain, fatigue, stress, flatulence and duration of constipation (which should be not less than 7 days, according to the Bowel Function Index, BFI) [3].

OIBD is often poorly detected or underestimated and, as a consequence, addressed later in comparison to other adverse effects of opioid therapy. In clinical practice, the available guidelines are not always adequately considered and are sometimes incorrectly applied [4].

### The gastroenterologist’s point of view

The distorted understanding of drug-induced constipation symptoms often leads to the administration of high amounts of bran, resulting in the formation of faecalomas. Elderly and bedridden patients are still treated with Vaseline oil which, in the presence of impaired swallowing, could cause tracheal aspiration and severe pulmonary reactions. More suitable approaches include the administration of osmotics for faecal softening, eventually followed on the third day by stimulants or enemas. The aim should always be the prevention of rectal faecalomas and the subsequent adverse effects, such as agitation, anal spasms, overflow incontinence and occasionally severe ulcerations [5]. The benefit of integrating a physical examination with a digital rectal exploration is scarcely recognised. Furthermore, the concurrent and self-prescribed use of herbal products may affect ongoing therapies, causing abdominal pain and inducing electrolyte imbalances [6].

The interpretation of guidelines by clinicians is partly misguided by their difficulty in understanding the subjective and objective nature of constipation: as a consequence, its diagnostic framework is still undefined.

A *position paper* published in 2016 by the Nordic Working Group, explicitly advises the use of fibre [7]. The position paper produced by the European Pain Federation (EFIC) suggests the use of laxatives and the exclusion of alcohol derivatives and glucose compounds, while other non-pharmacological indications are not clearly defined [8]. The 2015 Irish guidelines prepared by the National Clinical Effectiveness Committee advise caution when considering a digital rectal examination in immunocompromised or thrombocytopenic patients for the risk of fatal infections [9].

### The palliative care physician’s point of view

A relevant factor is the scarce awareness of symptoms by the patients, mostly concerned about pain and their illness. Particularly in home palliative care, nurses play an important role through education, assessment of symptoms and identification of appropriate treatment.

The report about bowel function in medical records is normally scarce. For opioid-naïve patients, a diary reporting the frequency of bowel movements, stool consistency and the use of laxatives, before and after the prescription of opioid therapy, is advisable. This would allow the clinician to identify differences between the pre- and post-prescriptive phases and formulate a definitive diagnosis of OIBD.

An abdominal X-ray might be helpful in selected cases when physical examination is inconclusive, but it should be recognised that it is often uneasy to perform for patients involved in palliative care programmes, particularly in the home setting.

Equally important is to measure the efficacy of treatment and the need for possible changes. Despite the availability of validated measurement scales, these are rarely adopted in clinical practice and limited to clinic research [10]. An example is the BFI, typically based on three parameters [3]:

1. ease of defecation;
2. feeling of incomplete bowel evacuation;
3. personal judgement of constipation.

The BFI can be considered a valid tool for the patient’s subjective evaluation of OIBD. However, also the following should always be monitored and recorded:

- frequency of bowel movements. Although three bowel movements per week are the optimal frequency [2], a bowel movement every 3 days should be the minimum acceptable habit, provided that stool shape and consistency are normal, in absence of straining to evacuate and/or incomplete evacuation. Information on laxatives taken by the patient is needed.
- rectal tenesmus, often described by the patient as a painful spasm, accompanied by an unsuccessful urge to defecate which, if not detected, could lead the clinician to increase the opioid dosage, further worsening the constipation. Importantly, rectal tenesmus must not be confused with the feeling of incomplete bowel evacuation.

To integrate the BFI with other items, a revalidation process is needed, which will require a rigorous scientific approach.

### The gastroenterologist’s point of view

Other parameters useful to address OIBD are the consistency and form of faeces, evaluated using the Bristol scale and the Rome criteria [11]. A patient with separate hard lumps and increased consistency is definitely experiencing a slowed intestinal transit.

Essentially, quick and replicable tools are needed, easily understood by patients and caregivers, to allow the assessment of the baseline condition and adequate ongoing clinical monitoring.

### The palliative care physician’s point of view

The most recent guidelines include indications often unfeasible for fragile patients in a palliative care setting, such as increased physical activity or specific dietary regimes [12]. Ensuring an adequate intake of liquids can be difficult, so the prescription of osmotic or softening laxatives is often problematic, and too frequently, there is a tendency to intervene with enemas and manual evacuation. These procedures are invasive and potentially painful. Such issues are particularly critical in the home setting, where the care burden lies mostly on the caregiver.

Nevertheless, specific recommendations for the management of constipation in palliative care setting have been made [13].

## Cancer versus non-cancer chronic pain

The issue of OIBD cannot be solely associated with the underlying disease, since opioids are used by a wide variety of patients, not only for cancer pain treatment. It is understandable that its psychophysical impact is greater in cancer patients, especially in those receiving palliative care, but the condition of patients under chronic opioid therapy for musculoskeletal pain or neuralgias should not be ignored.

## OIBD prevention or symptomatic treatment?

The onset rate of OIBD ranges between 2 and 40% [14, 15], suggesting the high probability for patients under opioid therapy to develop constipation.

OIBD therapy includes several lines of treatment, defined in the most recent recommendations and guidelines [12, 16].

The main characteristic of OIBD is a delayed colonic transit. Opiates interfere with the release of acetylcholine at the level of inhibitory neurons of the myenteric plexus, resulting in an increase in the circular muscle cells tone. The measurement of opioid potency relative to morphine is based on this model of inhibition [17].

This consideration suggests that the prescription of opioid therapy should be associated from the outset with prevention of constipation and close monitoring of therapeutic efficacy every 3 days, at least in the initial phase of treatment. However, pre-existing conditions must be always considered.

A key issue is preventing faecaloma. An increased intake of fibre may not be able to stimulate motor activity and paradoxically would favour the formation of faecalomas. Osmotic laxatives are often considered as the first choice to soften faeces, but they are associated with several side effects including flatulence and nausea. Saline laxatives may trigger electrolytic disorders, and disaccharides can cause abdominal distension and flatulence [18].

Among osmotic laxatives, macrogol can be definitely recommended as a preventive therapy, as it is not associated with relevant side effects, except inappetence and nausea when administered at high dosages [19].

An algorithm proposed in 2005 suggested [20]:

1. macrogol;
2. macrogol plus a stimulant laxative (senna or bisacodyl);
3. association of three or more laxatives in case of non-responsiveness.

### The gastroenterologist’s point of view

As a preventive treatment, administration of macrogol should start from 8.75 g of polyethylene glycol (PEG) 4000 combined with electrolytes, dissolved in 125 ml of water (or corresponding dose/volume of other macrogol compound) once a day in patients without constipation, up to a maximum dose of 17.5 g dissolved in 250 ml twice daily. The higher dosages could cause undesirable effects, such as loss of appetite due to fullness and nausea. In such cases, the dose of macrogol should be halved or other drugs should be considered. The response to macrogol generally lasts 5–6 days at the most; for this reason, administration should be started early. In case of unsatisfactory response, a prokinetic agent is suggested. Metoclopramide should be avoided due to its action on the proximal gastrointestinal tract and its side effects [21]. Prucalopride is a selective serotonin (5-HT_4_) receptor agonist stimulating the peristaltic reflex [22]. Principal adverse effects are headache, abdominal pain and diarrhoea.

Lubiprostone and linaclotide exert a secretagogue action, promoting intestinal motility and facilitating bowel transit. Potential side effects are watery diarrhoea, dehydration and electrolyte imbalance [23].

Physical activity, within the limits of the patient’s functional reserve, and hydration are also of paramount importance in the prevention of OIBD.

## Should PAMORA be recommended as soon as possible?

The dose-dependent effect of opioids on bowel transit is a discussed topic [24, 25]. The sensitization of enteric mu-opioid receptors is already noticeable at low doses of opiates. Indeed, some guidelines recommend increasing the dose of laxatives in parallel with the dosage of opioids [4].

It is widely accepted that the use of two or more laxatives combining a softening and a stimulating action may cause unpleasant side effects, from abdominal pain and flatulence to nausea and vomiting. Consequently, patients may require switching to two or more laxatives or the administration of other drugs against such effects, further worsening their condition.

If preventive treatments have proven to be ineffective, opioids should be rotated and, if no favourable results are achieved, a specific pharmacological therapy should be initiated. The drugs should be characterised by rapid onset of action, manageability and ease of use by the patient [16]. Because the predominant actions of opioids on the gastrointestinal tract are mediated by mu receptors, the administration of specific peripherally acting mu-opioid receptor antagonists (PAMORAs) is advisable. Methylnaltrexone and alvimopan were the early drugs in this group but they were not approved for oral use in OIBD [3, 26]. Naloxegol (Moventig®, Kyowa Kirin), the latest PAMORA, has been recently approved as the first oral drug for OIBD [27].

An objective criterion should be established to determine when to initiate the pharmacological administration of PAMORAs. A timely start would not harm the patient and would be consistent with the terminological use of the OIBD acronym. This approach deviates from the current indications [28].

The pivotal trials of naloxegol showed that the response rate achieved with naloxegol 25 mg was 14–15% higher compared to placebo, whereas in the subpopulation with inadequate laxative response (LIR), the response rate difference between naloxegol 25 mg and placebo exceeded 20% and the number of patients to be treated due to non-response was halved [16, 29].

Therefore, a possible algorithm is shown:

- Macrogol (125 ml) in the preventive phase, with reassessment every 3 days;
- at the onset of constipation, macrogol at incremental doses (125 ml twice daily up to 250 ml twice daily), with assessment every 3 days, for 1 week;
- in case of no response after 1 week, at the maximum dosage of macrogol, the concurrent administration of stimulants, such as bisacodyl 10 mg/day [30] or senna 24–48 mg/day [31]. The efficacy of sennosides may vary depending on the composition of the microbiota and the amount absorbed. Bisacodyl is preferred, because it is not assimilated into the enterohepatic system and is not activated by the intestinal enzymes [30];
- after a further week, in case of no response and after an enema rescue therapy, start PAMORA therapy.

If a patient is constipated and had not preventively received macrogol, it should be prescribed at the maximum dosage along with opioid rotation [32].

Based on the previously reported timeframe, from the onset of constipation, 14 days of intervention with macrogol and stimulants should occur before starting with the PAMORA agent.

### The palliative care physician’s point of view

For advanced cancer patients and terminally ill, a timeframe of 2 weeks could be excessively long. Two distinct scenarios should be outlined before pharmacological treatment with PAMORAs:

- a 14-day waiting time for outpatients and patients receiving early palliative care;
- a 7-day waiting time for terminally ill patients, with limited life expectancy.

In patients with an extremely limited life expectancy and in case of inefficacy of laxatives, the maximum waiting time should not exceed 2–3 days.

The early use of PAMORAs should be considered as a strategy for reduced social care costs of OIBD.

## Naloxegol starting dose

In two pivotal trials in which patients were receiving oral morphine, two doses of naloxegol, 25 mg and 12.5 mg, were compared [29, 33]. Efficacy with the 25-mg dosage was achieved in both trials, while in one trial, the 12.5-mg dose was ineffective [29]. It is therefore advisable that naloxegol should be initiated at a dose of 25 mg, possibly providing a subsequent de-escalation when needed or in case of impaired renal function.

### The gastroenterologist’s point of view

Risks related to naloxegol therapy include diarrhoea and abdominal pain. In the KODIAC-08 study at 52 weeks, the incidence of abdominal pain was higher among patients treated with naloxegol 25 mg compared to those administered usual therapy (17.8% vs. 3.3%); pain intensity, however, was not specified [34]. Only 9 out of 534 (1.7%) patients treated with naloxegol discontinued treatment due to abdominal pain [34].

Patients on naloxegol therapy should be contacted every 3 days during the initial stage of treatment to evaluate drug efficacy and side effects. Administering the drug every 2 days if abdominal pain occurs is not advisable; the only possible solution is a dose reduction or discontinuation of therapy [35] and to consider the impact of such events on patients’ quality of life in comparison to the constipation they were experiencing before starting naloxegol.

Another frequent event to be monitored is the patient’s self-interruption of medication once evacuation has occurred. This phenomenon has been reported in 80% of the cases of non-efficacy or reduced efficacy [36].

## Expectations from naloxegol therapy

When should the therapeutic response to naloxegol be considered satisfactory? A clinician should not necessarily aim for a complete resolution of constipation, as well as to meet the challenging target of total pain relief following the intake of strong analgesics. Guidelines suggest that patient’s personal opinion should be considered when assessing the benefits of the therapies.

### The gastroenterologist’s point of view

One evacuation every 3 days with soft and not separate hard lumps should be considered an acceptable therapeutic objective. Failing to evacuate on a daily basis could cause patients to experience abdominal bloating and to autonomously take laxatives. When taking naloxegol, laxatives should be discontinued. In the event of abdominal bloating, the administration of absorbents such as silicone or charcoal is advisable. Probiotics may be prescribed in case of mild to moderate abdominal bloating [37]. Finally, a dietary intervention is also possible, reducing the consumption of fibres and fermentable carbohydrates [38]. Abdominal bloating occurs predominantly in the initial phase of treatment with naloxegol and decreases with time.

Besides diarrhoea, abdominal pain and flatulence, headache and nausea are two other adverse events associated with naloxegol therapy [16]. Headache may not necessarily be related to the drug but may be attributable to an inflammatory reaction to concurrent factors (pain, evacuation strain, etc.). Nausea is likely to be associated with activity on the receptor [15, 39]. Its incidence is nevertheless lower than that of other adverse effects and it is less impacting and easier to control.

### The palliative care physician’s point of view

In palliative care settings, the patient’s subjective judgement of the negative impact of constipation on quality of life is a key element that must guide clinicians in selecting therapy. Often, the more the evacuation frequency resembles that preceding the onset of the disease, the greater the patient’s satisfaction. In patients at an advanced stage of disease, the simplest preventive strategy against abdominal tension caused by therapies is the administration of probiotics, while absorbents could affect the absorption of other drugs, making the therapeutic management of the patient more complex.

Definitely, a bowel movement every 3 days would be an acceptable goal, provided that the patient’s quality of life is subjectively satisfactory. Patients can be grouped into three types:

- responder (evacuating three times per week);
- ultra-responder (evacuation frequency higher than three spontaneous bowel movements per week);
- non-responder (evacuation frequency less than two spontaneous bowel movements per week). If difficult evacuation persists, reducing the naloxegol dose is not recommended.

### The gastroenterologist’s point of view

In the absence of a response, it is advisable to give macrogol at the maximum recommended dose (250 ml twice daily) and, on the third day without evacuation, administer a stimulant (bisacodyl or senna).

If a patient is a responder, naloxegol can be continued for as long as the patient is receiving opioids. To date, available data on the use of naloxegol do not extend beyond 52 weeks [34].

The Board agree on the definition of four clinical scenarios:

1. Patients responding to naloxegol in the short term: dose maintained and interruption of the treatment contraindicated. No tolerance of mu receptors has been observed at enteric level [40]. It is advisable to monitor renal function by evaluating creatinine levels [40, 41]. Given the lack of intermediate products, the substance is nontoxic and treatment can be considered safe in the long term. Drug metabolism via the cytochrome P450 system implies some warnings for patients taking the related inhibitors [40].
2. Patients responding to naloxegol in the short term and on a regimen of opioid rotation: consider a reduction of the dose and monitoring.
3. Patients responding to naloxegol in the short term and suffering from abdominal pain and bloating: consider a reduction of the dose and monitoring.
4. Patients not responding or not adequately responding to naloxegol in the short term: not advisable a dosage increase from 25 to 50 mg/day, as it would result in an increase in adverse effects, without leading to further improvement in efficacy [33].

## Cyclic or long-term therapy?

The Board unanimously agree that the naloxegol dose of 25 mg/day should be maintained, even if the patient reports an evacuation frequency higher than three bowel movements per week. A time-limited discontinuation could provide an indication of therapeutic efficacy in a responsive or ultra-responsive patient.

### The gastroenterologist’s point of view

The occurrence of a rebound effect in the cases of naloxegol discontinuation should be ascertained; unfortunately, such investigations are lacking.

### The palliative care physician’s point of view

It has been observed that for hospice patients with the low Karnofsky index, bedridden or receiving parenteral nutrition, the response to naloxegol is lower than for more active patients with a higher performance status [42].

The naloxegol dose de-escalation could be considered in the event of adverse effects or based on patient preference and during opioid switching. In contrast, naloxegol discontinuation should be considered when opioid therapy is discontinued, even in the case of persisting constipation. Particularly for non-cancer pain, if constipation persists after discontinuation of opioids and naloxegol, another cause must be sought.

Indeed, two different scenarios can be defined:

- in subjects with OIBD where opioids are administered for cancer pain, the duration of therapy with naloxegol should consider the prognosis of the disease. In responsive patients with a reasonable prognosis but with swallowing difficulties, the drug can be administered as a crushed tablet mixed in water and given orally or even via a nasogastric tube if already inserted for other therapeutic purposes.
- in subjects with OIBD where opioids are given for non-cancer pain, attempts to reduce and/or discontinue the opioid should be associated with periods of PAMORA discontinuation.

## How to manage opioid therapy?

Alongside naloxegol therapy, a patient with OIBD should have already undergone opioid switching. The selection of the opioid is based on the clinician’s judgement and not on therapeutic protocols, which have never been definitely established [43]. Early or preventive opioid switching is probably unnecessary and should only be considered for refractory or resistant patients.

The Board highlight the feasibility of using naloxegol in case of opioid switching to oxycodone plus naloxone (Targin®, Mundipharma Pharmaceuticals), or in case the patient experiences OIBD while already under this opioid. The association of naloxegol with oxycodone plus naloxone is generally not contraindicated [44]. An increase of the dose of naloxegol to 50 mg is not supported. Alternatively, in the absence of data, the Board suggest discontinuing naloxegol whenever oxycodone plus naloxone is prescribed in the opioid switch. It must be considered that the effects of equivalent doses of oxycodone and oxycodone plus naloxone do not always overlap, and cases of overdose or insufficient analgesia resulting from switches from one formulation to the other have been reported [45].

In summary:

- in case of oxycodone plus naloxone, naloxegol should be discontinued or not prescribed;
- in case of a switch from oxycodone plus naloxone to only oxycodone, naloxegol should be associated if OIBD occurs.

## Conclusions

OIBD is an important and common problem in the context of pain medicine and palliative care. The treatment of OIBD should be managed by a multidisciplinary team, and it is advisable to involve general practitioners, because patients on opioid therapy very often receive both hospital and home care. Anyway, the awareness and perception of this problem is still poor among health providers, despite the publication in recent years of guidelines and consensus conferences. We are aware that all the statements included in this text are expression of the opinions and personal experiences of a multidisciplinary Italian Board, which was constituted with the aim to offer a pragmatic and feasible approach to a heavy problem that affects many suffering and frail patients. Clinical research and practice jointly advance towards optimising the care of painful patients treated with opioids. PAMORAs are a valid and scientifically supported therapeutic aid for the treatment of OIBD. Among them, naloxegol is a promising drug, for which an increasingly widespread use is expected. The following would be desirable:

- studies on the duration of naloxegol therapy and on the effects of its discontinuation;
- studies on the efficacy of naloxegol after a dose reduction to 12.5 mg/day;
- studies on the association of naloxegol with laxatives in non-responsive patients, as suggested by the EFIC Scandinavian guidelines [7];
- pharmacoeconomics studies to assess the cost-benefit ratio associated with the strategy of anticipating the drug treatment with PAMORAs.
